# Similarities and differences between two well-performing healthcare systems: a comparison between the Israeli and the Danish healthcare systems

**DOI:** 10.1186/s13584-022-00524-x

**Published:** 2022-02-28

**Authors:** Daniel Kaminski Rotenberg, Brendon Stewart-Freedman, Jes Søgaard, Shlomo Vinker, Amnon Lahad, Jens Søndergaard

**Affiliations:** 1 The Research Unit for General Practice, Department of Public Health, University of Southern Denmark, Odense, Denmark; 2 Medical Center, Clalit Health Service, Tel Aviv, Israel; 3 Interdisciplinary Centre on Population Dynamics (CPop), University of Southern Denmark, Odense, Denmark; 4 Department of Family Medicine, Sackler Faculty of Medicine, Tel Aviv University, Tel Aviv, Israel; 5 CMO, Leumit Health Services, Tel Aviv, Israel; 6 Hebrew University, Jerusalem, Israel; 7 Department of Family Medicine, Clalit Health Services, Jerusalem, Israel; 8 Department of Public Health, University of Southern Denmark, Odense, Denmark; 9 Steno Diabetes Center Odense, Odense, Denmark; 10 Danish Aging Research Center, Odense, Denmark; 11 KI, Family focused healthcare research Center (FaCe), Odense, Denmark

**Keywords:** Denmark, Israel, Family Practice, Comparing, Health Economy

## Abstract

**Background:**

Denmark and Israel both have highly rated and well-performing healthcare systems with marked differences in funding and organization of primary healthcare. Although better population health outcomes are seen in Israel, Denmark has a substantially higher healthcare expenditure. This has caused Danish policy makers to take an interest in Israeli community care organization. Consequently, we aim to provide a more detailed insight into differences between the two countries’ healthcare organization and cost, as well as health outcomes.

**Methods:**

A comparative analysis combining data from OECD, WHO, and official sources. World Health Organization (WHO) and the Organisation for Economic Co-operation and Development (OECD) statistics were used, and national official sources were procured from the two healthcare systems. Literature searches were performed in areas relevant to expenditure and outcome. Data were compared on health care expenditure and selected outcome measures. Expenditure was presented as purchasing power parity and as percentage of gross domestic product, both with and without adjustment for population age, and both including and excluding long-term care expenditure.

**Results:**

Denmark’s healthcare expenditure is higher than Israel’s. However, corrected for age and long-term care the difference diminishes. Life expectancy is lower in Denmark than in Israel, and Israel has a significantly better outcome regarding cancer as well as a lower number of Years of Potential Life Lost. Israelis have a healthier lifestyle, in particular a much lower alcohol consumption.

**Conclusion:**

Attempting to correct for what we deemed to be the most important influencing factors, age and different inclusions of long-term care costs, the Israeli healthcare system still seems to be 25% less expensive, compared to the Danish one, and with better health outcomes. This is not necessarily a function of the Israeli healthcare system but may to a great extent be explained by cultural factors, mainly a much lower Israeli alcohol consumption.

## Key messages

- Israel has better health outcomes with lower healthcare expenditure, compared to Denmark.
- Israel has a different organization of its healthcare system, and a younger population, with a healthier lifestyle.
- Israel’s younger and healthier population and lower alcohol consumption may play a large role in explaining its better health outcomes, and lower healthcare expenditure.

## Background

This paper seeks to shed light on a subject of debate in the Danish healthcare community, about the balance of financial control between, primary care and the hospitals. The debate was triggered by the observed lower cost and better health outcomes seen in Israel, who has a different organisation in this area. Opinions vary widely between directly implementing features of the Israeli healthcare system due to the much lower cost and better outcome seen in Israel, and entirely dismissing the matter, attributing the observed Israeli advantages to differences between the two countries not related to the healthcare systems organisation. We aim to provide a more detailed picture of the two healthcare systems. Specifically, we addressed the points that debaters considered responsible, for the lower Israeli cost and better health outcome. These included differences in the number of community-based secondary care medical specialists, where it was suggested that better access to these specialists could improve community-based care. We use the term community-based secondary medical care here, in referring to community-based specialists other than General Practitioners (GPs). Another point of interest in the debate was the difference in health insurance coverage and co-payment, which may influence patient choices. Regarding healthcare expenditure, the Danish debate has focused on the absolute cost and the percentage of gross domestic product (GDP) spent on healthcare, disregarding the different age distribution, and without regard to purchasing power parity (PPP). While the Danish debate did include a discussion of the differences in the populations and the health risks and health outcomes, it was lacking in detail and included several misconceptions, and did not include healthcare quality indicators.

In Denmark, the remuneration of GPs is subject to national negotiations every few years, between the trade union of GPs and the national association of the five Danish Regions. Delegations with representatives from both these organisations do educational tours together, selecting countries with healthcare systems with potential to inspire; the tour of 2020 was to Israel. Both before, and after this tour, elected officials, scientists, and policy makers from both countries visited each other’s countries, and there was an intense debate among the Danish leaders and healthcare professionals, on what to learn from the apparently cheaper and better Israeli healthcare system [1–4]. This debate prompted us to provide a more in-depth description, and thereby offer the possibility of a better-qualified interpretation of the differences in the overall healthcare costs and results.

Both Denmark and Israel are known to have effective tax-funded well- integrated nationwide healthcare systems, covering the whole population, with strong primary healthcare sectors and universal digital medical records [5–15]. However, the two healthcare systems have important differences in performance and in their organization, with apparently better outcome at lower total expenditure seen in Israel [5, 16]. While both systems have drawn international attention, Israel is especially noted for its good health outcomes at modest expenditure [1, 2]. See “Appendix 1” for detailed information about the differences between the two healthcare systems.

## Material and methods

Our reference for this study is the six areas that the World Health Organization (WHO) has defined as the building blocks of a healthcare system (service delivery; health workforce; information; medical products, vaccines and technologies; financing; and leadership and governance/stewardship) [17]. We have chosen to focus on service delivery, health workforce, financing, leadership and governance/stewardship. We also investigate the health risks and the health outcome of Denmark and Israel, considering demographic, administrative and cultural factors. We did not find other studies comparing just Denmark and Israel, but identified several studies, including both, as well as several other countries, with cross-country comparisons addressing relevant topics. None of these evaluated differences in inclusion and definition of long-term care. Only one study adjusted healthcare expenditure for the varying age distribution between countries [18]. In order to better identify relevant areas to explore and to appropriately direct specific searches, the Danish first author (DR) spent two weeks in Jerusalem, observing clinical encounters of Israeli healthcare workers, interviewing doctors, medical staff and healthcare managers. Additionally, telephone interviews were done with healthcare professionals and patients who had experience of the healthcare systems of both countries. The interviews were in-depth and semi-structured. Data was thematically analysed. Recruitment for this was through the Hebrew University of Jerusalem, social media groups for Danes in Israel, and Israelis in Denmark. In total, 51 interviews were conducted, and 19 healthcare professionals were accompanied in their daily work. One or more medical directors from clinics of each of the four Israeli Health Maintenance Organizations (HMOs) were interviewed, as were medical directors from Terem Urgent Care Medical Centers as well as the Medical Director of the ER at the Sha’are Tzedek Medical Center.

We examined the number of GPs and community-based secondary care medical specialists in the two countries by using OECD reports, as well as national reports. Using national guidelines and reports, we also explored the organisation of their cooperation. Similarly, we investigated the differences in health insurance coverage and co-payment using a combination of OECD numbers and national reports and guidelines.

We identified major differences in health risk factors between the two countries, such as diet, obesity, and alcohol consumption, but also factors which are known to surface in public debate with only minor differences, such as smoking and overweight. Furthermore, we explored administrative, demographic, and cultural differences.

Regarding expenditure we examined not just spending expressed as a percentage of GDP, but also expenditure in PPP adjusted US$, as we believe this better describes the purchasing power assigned. We also calculated these numbers both including and excluding long term care, as this is not standardized between countries. All these measures are presented as observed and for Israeli spending, age adjusted to Danish age structure. Regarding health outcomes we chose to focus on life expectancy, child mortality, cancer, and cardiovascular disease. The OECD database is our main source of information. It includes data and metadata for OECD member states and selected non-member states, who provide the primary data according to agreed data protocols. Data are assessed and validated by specific committees within the OECD. Regarding healthcare expenditure we use the numbers for 2016, which is the latest year on which Israel has yet calculated its long-term care expenditure. Even though OECD Health Statistics report expenditure data for calendar years 2017, 2018 and 2019, these numbers include provisional statistics, and for 2019 mainly estimates. We use 2016 data because they are less likely to be altered by later revisions. We made the calculations for 2017, 2018 and 2019 with the long-term care for Israel in 2016 carried forward and found that using later data hardly affected the ratios between the two countries’ healthcare expenditure**.** Our findings in the discussion should be considered in relation to the methods we applied; when healthcare expenditure is compared between countries, it is commonly expressed in two ways. One, is by means of total healthcare expenditure per capita, i.e., dividing the total cost of healthcare by the population number, and then converting to a common and purchasing-power corrected currency such as PPP US$. The other way is by presenting the total healthcare expenditure as a proportion of GDP. Market currencies are subject to fluctuations and do not necessarily adjust for general price differences between the countries. Therefore, health care expenditure per capita is usually measured in PPP as this adjusts for differences in general price levels between the countries.

When comparing healthcare expenditure between countries, we assume that healthcare is defined similarly in those countries. Eurostat, OECD and WHO have developed a common set of methods defining healthcare and measuring healthcare expenditure [19]. Even using these methods, known as A System of Health Accounts, caution must be exercised in individual comparisons, since definitions of long-term care as either healthcare or social care continue to vary between countries [20]. Therefore, we compared healthcare expenditure with and without long-term care for both countries.

Usually, healthcare expenditure is compared without standardising or adjusting for demographic factors such as age; this contrasts with the way in which mortality and morbidity is compared. If we compare healthcare expenditure between countries to illustrate financial burdens, we should not adjust for age distribution. If the purpose is to compare the efficiency of healthcare delivery, it is necessary to adjust for age distribution since we know that national healthcare expenditure increases by the proportion of elderly in the population [21].

Healthcare expenditure in different countries is compared by percentage of GDP or by PPP adjusted per capita expenditure. Percentage of GDP is used more often, because as a proportion, it does not need to be adjusted over time due to inflation, cost of living, etc. The number “percentage GDP” directly represents how big a share of the country’s GDP has been assigned to healthcare expenditure. However, PPP adjusted per capita expenditure, while requiring periodic adjustment, better reflects the actual investment made in healthcare, because it more directly represents the purchasing power assigned to the healthcare system. Since we focus on cost-effectiveness rather than prioritization, we therefore prefer comparison by PPP adjusted per capita expenditure rather than a percentage of GDP.

### Data analysis

We compared healthcare expenditure both with and without the cost of Long-Term Care for both countries. In international account systems [19], the term “Long-Term Care” covers nursing and residential care homes, home nursing and social services, e.g. home aid. Many countries, including Israel, categorize most of these services as Social Services, thereby excluding most of them from the healthcare expenditure, whereas Denmark consider most of these services to be part of the healthcare expenditure [20].

We used estimates of the health care expenditure’s age growth factor to the proportion of elderly (65+) population, to age-adjust Israeli health care expenditure to the Danish proportion of elderly (18.8%); the Israeli proportion is 11.2%. With an age growth factor of 3.1% the age adjusting factor is 1.26 [18].

## Results

### Leadership, governance, service delivery and the health workforce

Denmark has more doctors per capita than Israel, but the headcount for specific sectors may not be directly comparable, as individual Israeli doctors frequently divide their worktime between hospital and community-based care, a practice rarely seen in Denmark, see Table 1. This applies particularly to community-based secondary care specialists, where the headcount for Israeli community-based specialists is three times that of Denmark. Since many of these specialists work only part- time in community-based secondary care, the Israeli specialist capacity is not actually three times that of Denmark. In both countries, patients in central areas have better access to health services, and healthcare positions can be filled with more ease. Travel distances for medical care are generally shorter in Israel, due to the country’s smaller size and contiguous landmass in contrast to the Danish bridge-linked archipelago, but overall both countries have excellent infrastructure with short travel times to healthcare providers [22].

**Table 1:** Overview of the structure of the four Israeli health maintenance organizations and the Danish regions [23, 24]

Organizations	Family doctors total	Family medicine specialists (%)	Specialists of family medicine and/or internal medicine (%)	Non specialists working as GPs (%)	Employment method	Current political leadership
Israeli HMO Clalit	2707	39	55	41	Mostly salary	Apolitical, Socialist Trade union origin
Israeli HMO Maccabi	1276	37	65	31	Mostly independent GPs*	Apolitical, Independent, origin
Israeli HMO Meuchedet	810	15	35	58	Mostly independent GPs*	Apolitical, Independent, origin
Israeli HMO Leumit	528	9	9	67	Mixed	Apolitical, Nationalist trade union, origin
The Danish Regions	3.326**	100	100	0	Independent GPs*	political democratic elected council

^*^Independent GPs refers to GPs who are private contractors, and are renumerated by the public system

^**^The breakdown of the Family doctors in Denmark by Region is: Hovedstaden 1033; Midtjylland 798; Syddanmark 775; Sjælland 435; Nordjylland 285

In Denmark, all permanent residents are offered a designated General Practitioner (GP), who has obligations towards them regarding availability, follow-up for their medical conditions and responsibility for incoming correspondence and test results. For Israeli GPs it is similar, but with even stricter guidelines regarding the GP’s responsibilities for follow-up correspondences and test results, and with automated reminders related to quality indicators integrated into the digital filing system. Danish GPs perform primary paediatric, as well as obstetric and gynaecological care, while in Israel this is commonly managed by community-based specialists of these specialities. Israeli patients usually receive their primary care from GPs or for children, paediatricians. Referring to GPs we include Israeli paediatricians performing primary care. Israeli patients can obtain obstetric and gynaecological care, ENT (ear, nose, and throat), orthopaedics, and dermatological consultation without referral, but usually require referral for other specialities, depending on HMO.

The Danish GP are financially incentivized by procedural fees, which are also the Danish Regions main control mechanism. The Israeli GP’s work is monitored by quality indicators which their management have incentives to optimize. Danish GPs have no similar quality program. In both countries GPs have similar responsibilities for a list of patients, and have comparable workloads. While Israeli GPs are obliged to offer more evening consultations, Danish GPs have more urgent-care obligations. See “Appendix 2” for further information. 

Both Danish and Israeli GPs offer remote consultation, by means of video, text messaging or telephone, where photos can be sent as a supplement. In both countries all laboratory and imaging results and discharge letters are directly available to the patient’s GP. Both countries have implemented national standards to enable digital communication in-between hospitals and with other health care providers. In Denmark all GPs and community-based secondary care medical specialists can choose any compatible digital filing system. In Israel the GPs use the digital system of their HMO, which is also compatible with the national standard. Some Israeli GPs allow patients to communicate with them through the GPs private cellphone including SMS, WhatsApp and Messenger; this is very uncommon in Denmark.

In Denmark GPs are reimbursed with a combination of an unweighted unconditional capitation fee, usually comprising 25% of the GP’s income, and by consultation and procedural fees, making up the balance. Danish secondary care community-based specialists only receive consultation and procedural fees, whereas the public hospitals` doctors are all salaried employees, regardless of specialist status. Conditions and remuneration are negotiated every few years, resulting in nationwide contractual agreements, with little room for individualization. Danish community-based secondary care specialists are usually forbidden to work in the hospitals. Secondary care specialists who are able to acquire a private fee license can see patients in a community setting remunerated by the public health insurance but must cease working within the organizational framework of the hospitals within two years. Though they can apply for being allowed to continue working part-time less than 10% have currently been granted this. Danish GPs rarely perform clinical hospital work. Part-time employment is uncommon for both hospital- and community-based doctors in Denmark.

In Israel each of the HMOs contract with individual doctors on reimbursement, resulting in a wide selection of options, ranging from salary, the most common, to a selection of procedural, consultation and capitation fees. The most common of these among GPs, is weighted capitation requiring at least one visit per quarter, with no additional pay for further visits. If the patient sees other GPs, the quarters capitation fee is divided. Both GPs and secondary care physicians may make agreements with one or more HMOs and other employers, on how much and where to work.

Israelis pay for more health services through supplementary private health insurance and through out-of-pocket co-payments. In Denmark most medical service is free at the point of delivery, and co-payments apply mostly for non-medical services. Danish private healthcare insurances cannot buy or expedite procedures in the public system but are intended for Denmark’s much smaller private healthcare sector. Israel has a large private healthcare sector, where the patients use of private healthcare insurances is integrated into the public system. See “Appendix 3” for further details about the above, Covid-19 handling and GP income and job satisfaction, and Table 2 for more about service delivery and the health workforce.

**Table 2:** Service delivery and health workforce

Subject	Israel	Denmark	Source
Number of Doctors per 1000 population	3.2	4.2	[25]
Number of GP’s per 1000 population	0.8	0.99	[26]
GPs to community-based secondary care specialist ratio, head count	0.97	3.05	[12, 27]
Total Private Health Insurance Coverage*	84.1%	34.9%	[15]
Out-of-pocket co-payment shares of current expenditure	21.1%	13.8%	[28]

^*^As mentioned in the text, OECD excludes non-active supplementary insurance policies, that are popular in Denmark

### Financing

Our health care expenditure reports are summarised in Table 3. Comparisons depend completely on our adjustments, i.e., with or without long-term care, and with or without age adjustment. This is due to the Israeli health care expenditure including only a small fraction of the long-term care expenditure included by Denmark, and because Israel has a younger population [5, 19, 20]. Furthermore, since the Danish GDP per capita is 37% larger than the Israeli GDP, the difference in health care expenditure as a proportion of GDP shrinks further. In fact, our health care expenditure comparisons range from 92% higher Danish expenditures to 9% lower expenditures compared to Israel, depending on the inclusion or exclusion of long-term care, whether the number is age adjusted, and whether it is expressed as PPP or as a percentage of GDP.

**Table 3:** Health care expenditure in Israel and Denmark, 2016: US$ PPP in 2016 and % GDP

	Israel	Denmark	Adjusted DK/ISR ratio	Israeli expenditure at Danish age distribution*	Ratio after Israeli age adjustment DK/ISR*
Total expenditure on health, US$ (PPP) per capita [29]	2520.3	4849.6	1.92	3175.6	1.53
Long-term care (Health), US$ (PPP) per capita [30]	199.0	1199.1	6.03	250.7	4.78
Total expenditure on health minus long-term care, US$ (PPP) per capita	2321.3	3650.5	1.57	2924.8	1,25
GDP, US$ (PPP**) per capita [31]	37,843.9	51,970.7	1.37	na	na
Total expenditure on health, percentage GDP [32]	7.2%	10.1%	1.42	9.07%	1.11
Long-term care (Health), percentage GDP [30]	0.6%	2.5%	4.44	0.76%	3.29
Total expenditure on health minus long-term care, percentage GDP	6.6%	7.6%	1.16	8.32%	0.91

^*^Multiplied by 1.26 [18]

^**^GDP is converted to US$ by a different deflator (PPP) than is used for health expenditure. Therefore, expenditures in % of GDP can only be computed from expenditures in national currency units

### ***Demographics, health risks and health outcomes***

Israeli and Danish lifestyles differ, including in areas with significant health impact. We chose key numbers pertaining to diet, showing large differences especially regarding alcohol, but also sugar and vegetable intake, see Table 4. The number of overweight and obese people were similar for the two countries, while obesity alone was more common in Denmark. Smoking prevalence was similar for both countries. Life expectancy may appear similar, but Israel ranks high—11th and 12th—in the OECD for life expectancy in 65-year-old women and men respectively, and Denmark ranks low, 35th and 30th out of 38 [33]. Figures for cancer incidence, cancer survival rates [34, 35] and cancer mortality, are significantly better for Israel, compared to Denmark, while figures for cardiovascular mortality are more similar, albeit still in Israel’s favour. Potential Years of Life Lost is significantly higher in Denmark. While infant mortality is lower in Israel, mortality for older children is somewhat higher.

**Table 4:** Health risks and outcomes

Subject	Israel	Denmark	Source
Life expectancy at birth	82.9	81.0	[37]
Inf. Mort. Rate/100.000 birth	3.0	3.7	[38]
Mortality 5–14-year-old children /1000/year, 2018 numbers	0.9	0.5	[39]
Age-standardized cancer incidence per 100.000 persons	233.6	340.4	[34]
Potential years of life lost Per 100 000 inhabitants aged 0–69	3300	3900	[40]
Potential years of life lost Per 100 000 inhabitants aged 75 years old	3367.0	3926.3	[41]
Deaths from cancer Per 100 000 persons	171	230	[42]
Age-standardized cardiovascular Mortality/100.000	12.2	13.5	[43]
Cancer deaths attributable to alcohol	4.2%	7.5%	[44]
Annual alcohol intake in citizens above the age of 15	3.0 L/person	9.7 L/person	[45]
Supply kg. Sugar/ year	31.3/person	55.3/person	[46]
Supply kg. Vegetables/ year	156.2/person	100.9/person	[47]
Supply kg. fruit/ year	112.4/person	59.8/person	[48]
Obesity (BMI > 30)	14.7%	16.8%	[49]
Smoking% of + 15 years old	16.9%	16.9%	[50]

Regarding the OECD database Health Care Quality Indicators, some indicators were clearly in favour of one country, others showed fluctuating results over time, sometimes without a clear trend [36]. Overall performance was similar; see “Appendix 5” for more information on healthcare indicators and patient satisfaction. 

Israel is home to a more culturally and ethnically diverse population, with greater income variation, compared to Denmark. The GINI index is the accepted way of describing income variation. A method similarly scoring ethnic fractionalization and cultural diversity was also found [51] (Table 5); see “Appendix 4”. Denmark is a largely monocultural state, with 86% of the population being Danish, 9% being immigrants from non-western countries or their descendants (mostly North Africa, Middle East and Central Asia), and 5% being immigrants from western countries or their descendants [52]. Israeli demographics are complicated. While there can be controversy on how the population is classified, it is safe to say that around three quarters of the citizens are Jews, split between a small majority of Mizrahi Jews (those descending from refugees and immigrants from the Middle East) and with Ashkenazi Jews (those descending from refugees and immigrants from European countries) being the second largest group. There are also many smaller distinctive groups, such as Ethiopian, Yemenite and Georgian Jews. The vast majority of the Jewish Israeli population is born in Israel, and the cultural differences between the various groups wane, as families live there for generations, and start to intermarry. Today the most visible division is by religious affiliation. The ultra-orthodox Jewish community, which includes Jews of many ethnicities, is notable for its significant difference from mainstream Jewish society. The differences are cultural and economical, and in the general approach to health and to the healthcare system. The non-Jewish population is mostly Arab, and is also split along religious and ethnic lines, with a large cultural difference between the rural and urban population. The main groups are Arab Muslims that can be divided into urban, rural and Bedouin, Arab Christians, and Druze. There are also small groups of various other affiliations, such as Armenian Christians and Black Hebrew Israelites, as well as working residents from all around the world [53]. In both countries, citizens performing national service can choose to serve in the public healthcare system, instead of the armed forces, and both countries receive donations to the healthcare service. While both types of contribution are more common in Israel, the numbers are very small compared to the total healthcare budget. See Table 5 and “Appendix 6”.

**Table 5:** Population

Subject	Israel	Denmark	Source
GINI Index	38.9	28.2	[54]
Ethnic fractionalization and cultural diversity scores	0.526/0.246	0.128/0.128	[51]
Percentage of population > 65 years	11.2%	18.8%	[55]
Donations% of total health budget	2%	0.002%	[56, 57]
National service in healthcare	2976 persons	< 99 persons	[58, 59]

## Discussion

Comparing healthcare expenditure depends on how and what is compared. Comparing observed expenditure per capita, Danish healthcare expenditure is 92% higher than Israeli expenditure. We see in particular that the sub-category *Long-term care (Health*) differs substantially, and it is known that reporting standards differ between OECD member countries. Some countries register most long-term (or social care) expenditure to the statistics of social services while other countries register the same expenditure as long-term care for the elderly and the handicapped, and hence as health expenditure. We believe this to be the case for Israel and Denmark respectively. Therefore, it makes sense to subtract those expenditures for both countries. Subsequently health care expenditures are only 57% higher in Denmark compared to Israel. Part of this difference may be due to different age distributions in the two countries; Denmark has more elderly residents. A crude age adjustment makes the difference shrink to 53% (for total health expenditure) and 25% for health care expenditure minus long-term care. Health expenditure in different countries is compared either per capita in purchasing power parity (PPPs) US$ or as a percentage of Gross Domestic Product. Since Denmark has a 37% higher GDP per capita compared to Israel, the differences and ratios between the two countries’ healthcare expenditures shrink further when using percentage of GDPs as measures rather than PPP per capita. Total expenditure on health ratio shrinks from 92 to 42%, health care expenditure ratio (i.e., subtracting long-term care) shrinks from 57 to 25%. And age adjustment shrinks the ratio further, to 11% and -9% respectively. So, the comparative span for the two countries’ expenditure on health ranges from 92% higher to 9% lower in Denmark compared to Israel, depending on how and what is compared.

As described in the method section, we believe that a comparison in PPP is better than percentage of GDP, when considering cost efficiency. Our best assessment is that the 9% higher expenditure in Denmark only expresses the difference in prioritization of healthcare, and that cost-effectiveness is best considered by using the number of 25% lower cost in Israel, calculated by using PPP, age adjusting, and subtracting long term care.

Israelis have much better access to community-based secondary medical care, compared to Danes. Danish GPs have a broader scope of treatments and are exclusively specialists. While these two factors may somewhat outweigh each other, with regards to which healthcare system is better, they are both commendable, and should serve to inspire healthcare leadership in both nations. The differences in management, working conditions and quality monitoring also include many points that could serve as mutual inspiration. In Israel the dynamic pace of change in regulatory mechanisms may be appealing to Danish healthcare organizers. In Denmark the almost universal self-employment and resultant obligation, liability, and continuity of the GPs, regarding their patients could be interesting to Israeli GPs and patients. 

The Israeli patient has more choice of providers within the public system, particularly if co-payment and/or supplementary or private healthcare insurances are utilized. In Denmark almost all consultations by GPs are done by specialists in family medicine, except those done by younger doctors in training, but Denmark lacks a quality indicator program similar to that of Israel. Despite a shorter Israeli GP training program, only a little more than half of Israeli GPs are specialists, and of these less than a third are specialists in Family Medicine. The most marked difference, however, is that the four Israeli HMOs can compete for patients by delivering healthcare differently, while the Danish Regions are encouraged to similar healthcare delivery, and Danish patients usually treated in their home region.

Large economic disparity within a society can make it harder to provide equitable care to that society. The larger the cultural and ethnic differences are in a society, the harder it is to provide equitable care to that society. The larger Israeli economic disparity, together with the more widespread requirement for co-payment for medical services and supplementary health insurance in Israel, may be a challenge to equitable healthcare. This may worsen Israeli health outcomes if patients seek healthcare later, leading to more advanced disease at the time of treatment initiation, which may increase cost. However, it might also lead to reduced cost if the delay is so severe that treatment is too late. We do not believe that this latter scenario is common for either Denmark or Israel, as both have a low total cap on co-payment and neither has co-payment for hospital treatments [60]. The greater ethnic and cultural fractionalization in Israel can present challenges to the Israeli healthcare system, which must adapt to a greater number of different approaches to health, compared to Denmark. This may lead to decreased health or increased cost [61, 62]. Hence, we believe that the better Israeli health outcomes and lower cost, is despite a greater economic, cultural, and ethnic disparity.

The Danish system, based on geographical regions, is intended to allow a high nationally standardized level of care and organization, despite lower population density, dispersed in a bridge and ferry linked archipelago. Israeli population is more concentrated, and situated in a continuous landmass, so Israel does not need to geographically fragment its healthcare system to the same extent to provide a better level of managed care and organization. For both countries central areas can recruit sufficient healthcare staff with more ease, and thereby better offer the required level of care. In Israel this refers to the coastal plain between Haifa and Tel-Aviv together with the Jerusalem area, where most of the population reside. Danish population centres are more dispersed with greater travel distances in between them. The distribution of Danish healthcare facilities, and the provision of sufficient patient flow to make these facilities economically viable and able to maintain expertise, has resulted in stricter control of both the location of healthcare providers, as well as patient choices.

Danish accountability for both finance and delivery of health services is more political, with elected officials bearing responsibility in elections. While officials are bound by the financial frame set by the ministry of health, they do have some degrees of freedom. Regional election results follow national voting patterns, but may differ somewhat, depending on which regional politicians are running, and what their policies are. Within the different parties, good performance of individual regional healthcare politicians can further their career, thus being a strong motivator for providing good leadership in the health politics of the Regions. As well as personal differences between the candidates, there are differences in the political parties’ approach to healthcare policy. These include placement of healthcare providers and cooperation with private healthcare actors.

In Israel there is competition for patients among the HMOs, and patients can change HMO due to healthcare services being delivered differently. This leads to more rapid changes in the HMOs, often causing other HMOs to adopt similar changes in order to better attract patients. Thus, Israeli healthcare has some of the traits of a free market, including the self-improving mechanism resultant of the HMOs being pitted against each other, but may also entail the drawbacks of populism and resources used on advertising.

We asses that donations and national service in the healthcare system do not significantly impact the difference in healthcare expenditure and outcomes, between Denmark and Israel. While donations and national service in the healthcare system can be assumed to decrease national healthcare spending or better outcomes, the total amount for both countries is low, though with marked differences. Donations are much less common in Denmark, but account for 2% of the total healthcare budget in Israel. Even these small numbers can be misleading, as donations can incur long-term running expenses for acquisitions that might otherwise not have been made. Regarding national service, even the higher Israeli rate is very low compared to the total number of people employed in the healthcare system, and since almost all the draftees are untrained, the impact may be insignificant.

In Israel, Potential Years of Life Lost is lower, Longevity is higher, and Survival from Cancer is better. Is this partly due to the healthcare system? Maybe, since Israel has a different organization in its primary healthcare, but first the higher Danish alcohol consumption should be recalled. The better Israeli outcome and lower expenditure could be partly or mostly due to other factors, especially the Israeli citizens’ lower alcohol intake. Israel also has a lower sugar consumption, higher vegetable consumption and a generally healthier diet [5, 63], together with a more hilly terrain, a warmer climate and less precipitation, all factors that improve health outcomes and decrease healthcare expenditure, albeit on a smaller scale [64, 65].

Regarding the healthcare quality indicators, it is not clear which country is better. Registration of the data is influenced by differences in organisation and medical culture. For instance, a COPD or asthma patient with a severe exacerbation, will receive the same care at the hands of doctors of same level of training; in Denmark this will be in the framework of a hospital Emergency Room (ER), while in Israel this will often be in an urgent care community setting, and thus not be registered as a hospital admission.

## Conclusion

Is the Israeli healthcare system better organised, and therefore cheaper than the Danish Health Care system? While healthcare expenditure is lower in Israel than in Denmark, the latter ranks better in other areas, such as less economic barriers to secondary and tertiary medical care. Furthermore, the gaps in expenditure are minor when adjusted for age and long-term care. While the organisational differences between the Israeli and Danish healthcare systems are interesting, the influence of cultural factors on healthcare expenditure and outcomes, particularly the more than three-fold higher Danish alcohol consumption, should not be underestimated. Our best assessment is that this may explain most of the 25% lower cost, that remains after adjusting for age and excluding long term care, as well as explain most the observed differences in health outcomes. A large alcohol consumption is known not only to increase the incidence and severity of most diseases, but also to diminish the therapeutic response and prolong treatment, all factors that may contribute to greater healthcare expenditure and poorer outcomes. While we believe that our paper should cause Danish policy makers to focus on decreasing Danish alcohol intake, further analysis should also investigate what the two countries could learn from each other’s organisational structure, as we find both to have many unique and interesting features.

## Data Availability

Upon request from the author.

## Appendix 1

In theory the Danish healthcare system is a Beveridge system, and Israel follows a Bismarck system. The Israeli health system is funded by a mix of general taxes, as in Denmark, and earmarked taxes, which is different from Denmark. The main difference is in the leadership and organization; the two healthcare systems are different regarding the division of work between sectors, and the distribution of funds for hospital and community-based care [66]. Israel has four competing publicly- funded Health Maintenance Organisations (HMOs) between which citizens can choose freely. HMO membership is not geographically determined, and each HMO must provide service throughout the country. The Israeli HMOs are self-owning non-profit entities that are professionally managed and perform community-based healthcare and arrange hospital services for their members. Israeli HMOs are charged by the hospitals for services rendered to their members. Their funds are pooled and redistributed through a capitation-based risk-adjustment formula, as in Denmark. They receive funds equal to almost half of Israel’s total healthcare expenditure. Israeli public hospitals are variously owned and run by the State (50%), by Clalit the largest HMO (35%) and by a small number of private organizations. The Israeli HMOs must offer the same basket of services as each other but compete on delivery. The government defines the basket of services**.** Israeli national health law determines what is in the basket; the law is made by the Knesset and reviewed annually. The basket is interpreted by each HMO under the arbitration of the Ministry of Health. The total funds that the Israeli HMOs receive are allocated according to demographic differences, and whether the area in which they live is central or peripheral.

Denmark's five Regions are geographical, political, and administrative entities with their primary responsibility being to manage all public healthcare activities in Denmark. Danes have no choice of which region they want as payer agency, as this is determined fully by where they live. The Regions are operated by civil servants whose executive level is supervised and governed by locally elected politicians, with only the chairman of these being remunerated for fulltime service [67]. Denmark has no defined basket of services, but nevertheless a clear consensus exists in the healthcare system as to which services to offer. This is primarily based on the medical societies of the different specialities, though in the case of community care it is also significantly influenced by the national agreements on which fees can be charged for different types of consultations and procedures. While the Ministry of health may intervene and prohibit or mandate certain treatments, this is uncommon, and is usually reserved for expensive hospital treatments.

All public healthcare is governed by the democratically elected councils of the five Regions and the similarly elected councils of the 98 municipalities. The regions are responsible for both hospital and community care. The term community care is used here to describe primary and secondary medical healthcare, delivered outside the organisational framework of the hospitals. The municipalities do not employ doctors for clinical work, and their health-related responsibilities are mainly home-based nursing care, long-term nursing care, non-specialized rehabilitation, and preventive care. Though Danes can select healthcare providers outside their home region, hospitals can refuse these patients due to lack capacity, save urgent and emergency care. The community care of the municipality is usually performed in the patient’s municipality of residence. While Danes can seek a GP (General Practitioner) or community-based secondary care medical specialist throughout the country, usually local healthcare services are utilized. Thus, almost all healthcare of a Danish patient is within their home region, and for municipality-based services, within the municipality of their residence.

The Regions must all provide the same services and are funded by age-adjusted grants from the State, and in turn the Regions run the hospitals, and provide them with almost all their funds and provide almost all community care by contracting with private healthcare providers such as GPs and the community-based medical secondary-care specialists.

Many Israeli patients have supplementary health insurance, which can allow them to shorten waiting time and gain access to a wider choice of experienced specialists. In Denmark only ENT and ophthalmologists are directly accessible, and even here appointments can be hard to get without a referral.

Both the Israeli HMOs and the Danish Regions, fund more than 95% of the activities in General Practice (including paediatric primary care in Israel). Most of the functions that take place in community-based secondary medical care in Israel, are found in the Danish hospitals as ambulatory services in fields such as Internal Medicine and Surgery.

## Appendix 2

In both countries GPs are responsible for a list of patients. In Denmark a GP must have at least 1600 patients before being permitted to close his list. Danish GPs can apply for a reduced list size, but less than 5% have currently been granted this. In Israel salaried GPs have an agreed upper norm for the list they can be required to service, with a progressive reduction, considering age. For a young GP it is 1530 patients, and for 50-year-old 1230. The independent Israeli GPs list is subject to individual negotiation but is usually smaller. In Denmark the GP is obliged to offer urgent consultations the same day and offer ordinary consultations within 5 weekdays. This is not strictly enforced regarding ordinary consultations, and may also not apply in full for neither urgent or ordinary consultations if the GP has deemed the initial telephone assessment sufficient. In Israel each HMO decides how fast their GPs must offer ordinary consultations, currently within two or three working days. While in principle the Israeli GP has an obligation to see urgent patients, unlike in Denmark, these patients have the option of visiting walk-in urgent care centres. The Danish GPs usually work in an egalitarian leadership structure, where the clinic doctors are partners, and no other leadership structure is in place. Regulation is by law and contractual agreement. Conflicts are resolved within the union structure, or via the judicial system. In most Israeli clinics one of the GPs is appointed as manager by the HMO and supervises the others. Access to the Israeli GPs patient files is possible for doctors in their HMOs management. Solo clinics, with just one doctor, are still common in both countries. In Denmark almost all GPs are independent contractors, who hire and supervise their own staff, select equipment, and choose the clinic site within the area connected to the fee license. The Danish GPs are contractually obligated to cover their patients’ needs within 08-16 on weekdays, if not themselves, then by arrangement with other GP clinics. The Israeli GP is usually an employee, and even if independent, has a hierarchy of managers above him, and usually works at an HMO site. The staff of the Israeli GP clinics is usually hired by the HMO, but supervision of them is joint with the GP manager of the clinic. Evening consultations are offered in both countries, but more so in Israel. The Danish GP has more control of the appointments that the patient can book at the clinic than his Israeli counterpart. Israel *prefers* its GPs to be specialists, particularly in Family Medicine, though many GPs are not specialists in any field. In Denmark all GPs *must be* specialists of Family Medicine. The leadership of the Danish regions is political, while the Israeli HMOs are no longer politically affiliated. Refer to Table 1 which also includes a breakdown of the different reimbursement methods used.

## Appendix 3

The average Danish GPs personal income in 2017 was 189,000 US$ before tax. As the Israeli GPs have better opportunities for reduced workload a comparable number is hard to find, but they usually earn between 122,000 US$ and 215,000 US$ depending on seniority and hours. Overall medical salaries and price levels are similar for the two countries [68–71]. We did not find reliable comparable data for GP job satisfaction, but one cross-country comparison including Denmark, but not Israel, put Denmark on top, out of 34 countries [72, 73]. Israelis pay for more of their health services themselves, through supplementary private health insurance, which many more have compared to Danes, and through out-of-pocket co-payments, accounting for 22% of total expenditure in Israel and about 14% in Denmark [5, 66, 74]. In Denmark almost all medical service is free of charge at the point of delivery, and co-payments only apply for prescription medication, dental care, and other non-medical services. In both countries, GP consultations and medications given to admitted patients are free of charge. In Israel there is co-payment for imaging, hospital, and community-based secondary care specialist consultations, as well as for medications and dental care. The level of these co-payments depends on what supplementary insurance the patient has. In Denmark, all imaging and hospital service in the public system is free of charge. For the 99.7% of the Danish population, who have chosen the health plan called “Group 1”, consultation with community-based secondary care specialists is also free of charge. The remaining 0.3%, who have chosen the health plan called “Group 2”, have access to community-based secondary care specialists without referral, but must pay a fee for these visits, as well as for consultations with their GP, and do not have a GP formally responsible for them. Aside from that, they have the same rights, and similarly do not pay for hospital and imaging services, and the results of all tests performed on them is still in the end, the responsibility of the doctor who ordered them [58, 69, 75–81]. In both countries consultations with psychologists and physiotherapy are subsidised.

The OECD numbers cited in Table 2 for total private health insurance coverage are problematic, as the OECD data in the database do not include Denmark’s largest private health insurance companies, due to the nature of their insurances (passive). If these were included, it would show that more than half of all Danes have supplementary health insurance. These, however, cannot buy or expedite procedures in the public health system, but are intended for Denmark’s much smaller private healthcare sector. In contrast to this, Israel has a large private healthcare sector, where the patients can use their private healthcare insurances, and where their use is completely integrated into the public healthcare system. In both countries GPs have had to adapt to the Covid-19 crisis by transferring consultations with potential Covid-19 infections to specialised centres. In Israel GPs have played a role within each HMO in motivating employees and patients to receive the Covid-19 vaccine. While Danish GPs have not played an official role in motivating health care employees and patients, they have been generally supportive of the vaccine program. In both countries, vaccination coverage is good.

## Appendix 4

*Ethnic fractionalization* is defined as the probability that two individuals selected at random from a country will be from different ethnic groups. *Ethnic diversity* is the magnitude of the differences between these ethnic groups; while Danes, Germans, Englishmen, Americans, Norwegians, and Swedes are of different ethnicities, the cultural differences between them are small.

## Appendix 5

The OECD database contains 60 Health Care Quality Indicators, of which 36 include data for both Denmark and Israel. Some of the indicators are sums of indicators already stated in the same dataset, and some are linked and unlinked versions of the same data. We reviewed all 36 datasets, concerning the last 10 years. We also examined other studies reporting on differences in mortality and morbidity in cancer and cardiovascular disease.

Israel scored significantly better in preventing hospitalization for Asthma, Chronic obstructive pulmonary disease (COPD) and Diabetes, while Denmark scored significantly better in preventing admission for Heart Failure and Hypertension. Regarding diabetics lower limp amputation, Denmark scored much better. While 30 day mortality from Acute Myocardial Infarction (AMI) was more similar, albeit slightly in Denmark’s favour, the reverse was true for stroke. Regarding hip fractures, Danes receive treatment faster. In-patient suicide, and suicide 30 days and 1 year after discharge, was more common in Denmark, but the over-representation by percentage is similar as Denmark has a higher suicide rate in general. Regarding excess mortality of patients with severe mental disorders, the rates are similar. Post-operative sepsis was much more common in Israel while obstetric trauma was more common in Denmark. Benzodiazepine treatment in elderly patients was much more common in Israel as was the use of antibiotics in general, with a much higher use of second line antibiotics in Israel.

Comparable data are hard to get, but patient satisfaction is very high in both countries, with marginally better scores in Israel. These results should be considered with caution, due to different survey methods, and may be even further influenced by cultural factors [82, 83].

## Appendix 6

As a workforce financed from outside the healthcare budget could potentially decrease public healthcare expenditure and increase quality, we chose to investigate this. Comparable figures for national service in the public healthcare system were only found for 2011, but we asses them to be representative for later years. While 2967 Israelis served their national service in the healthcare system, only 99 Danes served any form of alternative national service, and of these, an unknown smaller number served within the healthcare system. As shown in Table 5, in total the Israeli healthcare system receives 2% of its funding through donations, while the Danish healthcare system receives 0.002% of its funding in that manner.

## Abbreviations

WHO: World Health Organization
OECD: The Organisation for Economic Co-operation and Development
PPP: Purchasing Power Parity
GDP: Gross Domestic Product
GP: General Practitioner
GPs: General Practitioners
ENT: Ear, nose, and throat
HMOs: Health Maintenance Organizations
ER: Emergency Room
COPD: Chronic obstructive pulmonary disease
AMI: Acute Myocardial Infarction
